# An Account of the Chief Peculiarities of the Brunonian System

**Published:** 1799-04

**Authors:** 


### *An Account of the chief Peculiarities of the Brunonian Syſtem.*

At a time when every Univerſity in Europe and America, and practitioners in both Indies, are directing their attention, by encomiums or criticiſms, to the *Brunonian Syſtem*; when it has begun to be adopted, and openly avowed by many popular writers in this kingdom; it may not be thought unſeaſonable to lay before our younger readers, a conciſe view of its principles and peculiarities.

peculiarities. Though dignified by its admirers with the title of a ſyſtem, it will be obvious to the diſcerning reader, that there are many chaſms in it, in common with all other medical ſyſtems; and many errors, the correction of which will require much time and obſervation, if the fundamental principles of it ſhould be admitted.

The circumſtances attending the promulgation of Dr. BROWN's opinions, at firſt in Edinburgh, made the profeſſors and eſtabliſhed practitioners unite very generally in oppoſing them. They might have perceived, that the application of his ſyſtem to practice, was by no means ſo ſimple and obvious, as the young ſtudents, who firſt embraced it, imagined; and therefore they might have oppoſed it from an opinion of its dangerous tendency, as well as a diſapprobation of the conduct of its author. This opoſition however, in an Univerſity, which, ſince the death of Boerhaave, has dictated the medical opinions of Europe and America, contributed moſt effectually to diſſeminate and eſtabliſh the new doctrines of Brown. The Medical Societies of Edinburgh, inſtituted for the diſcuſſion of theoretical and practical ſubjects connected with Medicine, have an almoſt irreſiſtable influence over the opinions of its ſtudents. In order to defend his own diſſertations in theſe ſocieties, or attack thoſe of his cotemporaries, every member muſt acquire the ideas and phraſeology which prevail at the time; and that too at a period of life, when all forcible impreſſions remain indelible. It muſt be obſerved, that the members who attend theſe ſocieties, conſiſt almoſt entirely of young ſtudents, and the preſidents are always elected from among them; ſo that their debates are never over-awed by the preſence of profeſſors or eſtabliſhed practitioners. It is well known that the Society, called the Royal Medical Society of Edinburgh, were in the habit of diſcuſſing medical ſubjects, on the principles of the ſpaſmodic, or that ſince called the Cullenian theory, which they had acquired from the works of Hoffman, long before the Profeſſors themſelves had relinquiſhed the opinions of the Boerhaavian ſchool. It is alſo true, that during the latter part of the life of Dr. CULLEN, the Brunonian ſyſtem was adopted in all theſe ſocieties, though the graduates of that Univerſity were not allowed to publiſh their theſes upon any other principles than thoſe taught by the Profeſſors. The ſyſtem of Brown, adopted and diſſeminated, by at laſt 200 young men annually, from which number the ſurgeons and phyſicians of the navy and army are generally ſupplied, as well as the practitioners of the Eaſt and Weſt Indies, muſt neceſſarily in eight or ten years affect the opinions of the whole medical world. This was really the caſe; but perſons eſtabliſhed in the profeſſion were ſomewhat ſhy and backward in declaring their opinions, till DARWIN profeſſed himſelf to have been a Brunonian, even before he had heard of Brown's ſyſtem.

The learned were aſhamed to avow the opinions of PARACELSUS, before VAN HELMONT openly adopted them. This reluctance in the human mind, againſt being led by an individual, or being the firſt to join an innovator, appears to ariſe from the unwillingneſs of admitting a dictator, or from the ridicule commonly thrown upon an early apoſtate from eſtabliſhed opinions.

Since the publication of Zoonomia, the language and ſentiments of Brunonianiſm are become common; but what is remarkable, though by no means ſingular, on the occaſion, is, that a majority of the perſons who are become converts to the doctrine, are totally unable to recollect, when or how they were converted.

But it is our buſineſs to give an account of the ſyſtem, rather than of the means which retarded or promoted its promulgation.

The common opinion reſpecting life, or the vital principle in animals and vegetables, is, that it is entirely diſtinct from the organization of the body in which it reſides; that it is a ſeparate, independent principle, added to the body in ſome early period of its exiſtence, and which there continues unchangeable, and then leaves it at a late period, when it finds the habitation no longer tenable. Dr. Brown, on the contrary, conſiders life as an aſſemblage of actions or effects, which take place in the body, in conſequence of a certain prediſpoſition and exciting cauſes; and that the ſtate or quantum of the vital principle, or energy of the ſyſtem, is *perpetually varying.* Thus the abſtraction of heat and food may reduce the powers of life ſo low, that the hot bath, or a glaſs of wine, would be ſufficient to deſtroy the patient. On the other hand, a jail fever, in a few days, may ſo far diminiſh the vital energy, that a warm room, and a bottle of wine a day, may become neceſſary to preſerve life. In the former caſe, the prediſpoſition is ſaid to be morbidly accumulated, in the latter, exhauſted. This ſhort ſtatement includes the baſis of the ſyſtem; but, before we proceed to develope it further, it is neceſſary to explain a few terms, which are peculiar to the doctrine, or employed in a peculiar ſenſe.

The degree or ſtate of action, or vigour of the ſyſtem, or energy of the vital principle, which is preſent at any time, is here called

### EXCITEMENT.

It has been ſuggeſted, that Dr. Brown adopted this term, becauſe Dr. Cullen had rendered it faſhionable and familiar to the profeſſion, though he uſed it in a more limited acceptation. We rather ſuppoſe it was preferred on account of its implying here no particular hypotheſis.

That

That ſtate of the organization of the ſolids and fluids, which conſtitutes the prediſpoſition to *Excitement*, is denoted by the term

EXCITABILITY.

Some of Dr. Brown's followers, who were of opinion that the *Excitability* of the ſyſtem depended upon the ſtate of the muſcular fibre alone, employed the word *Irritability*, as ſynonymous with *Excitability*. But this is objectionable, as being founded on an opinion not generally received.

All thoſe powers, both internal and external, ſuch as the paſſions, heat, food, medicines, contagion, pain, &c. which, by acting upon the Excitability, produce Excitement, are included under the general name of

STIMULI.

This term is perhaps more objectionable than either of the preceding, on account of the enormous extenſion of its application. Stimulants and ſedatives were terms that had long been received as antagoniſts in a medical ſenſe. The annihilating one, and making it only a degree of the other, was a ſhock to medical language too great to be acquieſced in on a ſudden. Yet we know, that in the language of the profeſſion, heat and cold were formerly conſidered as antagoniſts, but now nobody doubts that they are only different degrees of heat.

The ſame error pervades medical language, when ſpeaking of the exciting paſſions; the effects of hope are often imputed to fear, which is only a different degree of hope.

If, for the ſake of avoiding the term *Stimuli*, Dr. Brown had uſed *Exciting Powers*, the abſurdity of exciting powers producing depreſſion, in however low a degree applied, would have appeared more objectionable than the generalization of the term. We ſhall ſoon ſee, that the different *degrees* or *intenſities* of ſtimuli, may often be ſubſtituted for moſt of the different genera and ſpecies of them, as well as thoſe ſuppoſed *antagoniſts*.

Having explained the chief radical terms peculiar to this ſyſtem, we ſhall next proceed to its developement with reſpect to the operation of ſtimuli upon the Excitability, in producing the various degrees of Excitement, upon which all health and diſeaſe are ſaid to depend.

1. The *Excitability* of the whole body, as well as of particular parts, is by Dr. Brown ſuppoſed to be in a ſtate of *perpetual variation*.

This variation depends upon the time and manner of application, of the internal and external ſtimuli employed.

To

To illuſtrate this fundamental poſition, we may inſtance the change which takes place in the progreſs of life, independent of accidental circumſtances. In the firſt days after birth, excitability of the primæ viæ is ſuch, that a few grains of manna will act as an operative doſe. During the firſt year, the healthy excitement of the ſyſtem may be ſupported by a milk diet; and it argues an abuſe of ſtimuli, if a glaſs of wine does not prove an exceſſive ſtimulus before the age of puberty. In advanced life it is well known, that the ſtimuli juſt mentioned, are far too feeble to produce any obvious effect or excitement. If theſe ideas were not founded in truth, there is no obvious reaſon why animals and vegetables might not be immortal.

The accidental circumſtances, which we have juſt alluded to, as moſt commonly producing variations in the excitability, are, the internal and external ſtimuli above mentioned. The changes, however, depend almoſt entirely upon the manner of applying them. It is not the rare or caſual operation of ſtimuli, which produces any permanently important variation in the excitability; but that which, frequently and regularly repeated, changes cuſtom into habit. This may be illuſtrated by referring to the effects of opium, tobacco, ſpirits, &c. upon perſons accuſtomed to the uſe of them. We may alſo advert to the various ſtates of the excitability at the commencement and during the progreſs of fevers; in perſons properly fed and clothed; and in the ſame perſons, when accidentally deprived of theſe comforts, &c. &c.

2. The degrees, intenſity, or ſum of ſtimuli, which act upon the excitability, and regulate the excitement or energy of the ſyſtem, ought to be conſidered in reſpect both of force and permanency. But, before we can ſpeak of the force or intenſity of the exiſting ſtimuli at any time, it will be neceſſary to obviate the inconſiſtency above alluded to, in calling thoſe things ſtimuli, or exciting powers, which produce ſedative or debilitating effects. If it were poſſible to exhibit any ſubſtance entirely void of heat; or to conceive a total abſence of internal ſtimuli during life; and if we had terms to denote theſe circumſtances, in various degrees of intenſity, which is obviouſly abſurd, and impoſſible, then might we employ the terms "*power of cold*," "*directly debilitating powers*," &c. without outraging the common acceptation of terms. The author of this ſyſtem has been accuſed of a want of preciſion in this reſpect.

No perſon can doubt, that an abſtraction of the cheerful paſſions, of heat, or of neceſſary food, may directly and immediately produce debility. This is the debility ariſing from deficient ſtimuli, and called by Dr. Brown *Direct Debility*. But as this ſtate of the ſyſtem is found to be more ſuſceptible of the operation of ſtimuli, than the healthy ſtate, it is inferred that the *exci-*

*tability*

*tability is accumulated;* ſo that *direct debility* and *accumulated excitability,* are employed as equivalent terms.

When the energy of the ſyſtem has been diminiſhed, or debility produced, in conſequence of the inordinate application of ſtimuli, as of joy, heat, voluntary motion, wine, opium, &c. this delibity, as being *conſequent* to unuſual *excitement,* is called *indirect debility,* or *exhauſted excitability.*

According to this ſyſtem, health, and continued vigorous action of the body, depend upon a due balance or proportion between the ſtimuli and excitability, ſo that the latter may neither accumulate nor be exhauſted for many hours together. It therefore follows, that all diſeaſe ariſes from a morbid accumulation or exhauſtion of the excitability, or from direct or indirect debility. And as two different degrees of excitement cannot poſſibly exiſt in the ſame perſon ſimultaneouſly, it is impoſſible that two different conſtitutional diſeaſes ſhould be preſent at the ſame time.

From this ſhort ſketch of the cauſes of health and diſeaſe, according to this ſyſtem, it will be obvious, that the preſervation of the former, or the cure of the latter, muſt principally depend on due application of ſtimuli.

If time and experience had reduced this to fixed rules, nothing would be wanted to the completion of the Brunonian doctrine. In order to explain this part of the ſubject to the younger claſs of our readers, we ſhall adduce a few inſtances.

If a perſon who had been confined for ſeveral years in a cold and dark dungeon, and fed on bread and water, were committed to our care, or cure, for the ſtate of his ſyſtem could not be that of health, though no ſpecific diſeaſe may be actually preſent; we ſhould not expoſe his eyes to the glare of the ſun, his body to the hot bath, his limbs to fatigue, or his ſtomach to fermented liquors. In the practice to be adopted, all are agreed ; but the Brunonian explains it in this manner:—The excitability being accumulated in ſo inordinate a degree, the ſtimuli to be adopted muſt not exceed thoſe uſually applied to a new born child, otherwiſe a fatal inflammation, or ſudden death, would enſue. But, if the ſtimuli of light, motion, and food, be applied at firſt in very low degrees, the excitability may be gradually brought down to the common ſtandard ; and of courſe become capable of bearing the ſtimuli uſually applied to healthy perſons.

If, on the contrary, we found a patient who had been affected for ſeveral days with jail fever, and reduced to as great a degree of debility as could be compatible with life, the whole profeſſion would agree in the mode of treatment; that is, in applying warmth, or bliſters, externally, and in giving brandy, wine, ſpices, opium, æther, bark, &c. in appropriate doſes, internally. The Brunonian juſtifies and explains his practice, by ſtating, that the excitability is ſo rapidly and inordinately exhauſted in theſe fevers, that an excitement compatible with the continuance of life, and reſtoration of health, can alone be produced and ſupported by the moſt powerful and diffuſible ſtimuli.

We perceive, then, that the cure of all diſeaſes, according to this ſyſtem, conſiſts in proportioning the ſtimuli to the degree of excitability preſent in the patient, till healthy excitement is reſtored.—As a general rule, we are adviſed to apply the ſtimuli in the inverſe ratio of the excitability, in order to produce the moſt ſalutary action, or excitement of the ſyſtem. Dr. Brown, and his adherents, explain this in the following manner.—They ſuppoſe any ſtate of the excitability, compatible with the continuance of life in the extremes, or with health in the middle of the ſcale, may be repreſented by the common numbers, from 1 to 19; and that the different degrees of ſtimuli which may be applied to it, to reſtore or preſerve health, may alſo be repreſented by the ſame numbers in the inverted order.—Thus,

| | Excitability, or Predisposition. | | Sum of Stimuli. | | Product, or Excitement. | | | |
|---|---|---|---|---|---|---|---|---|
| Accumulation. | 20 | —— | 0 | —— | 0 | death | A | Direct debility. |
| | 19 | —— | 1 | —— | 19 | —— | B | |
| | 18 | —— | 2 | —— | 36 | —— | C | |
| | 17 | —— | 3 | —— | 51 | —— | D | |
| | 16 | —— | 4 | —— | 64 | —— | E | |
| | 15 | —— | 5 | —— | 75 | —— | F | |
| | 14 | —— | 6 | —— | 84 | —— | G | |
| Health. | 13 | —— | 7 | —— | 91 | —— | H | Limits of health. |
| | 12 | —— | 8 | —— | 96 | —— | I | |
| | 11 | —— | 9 | —— | 99 | —— | K | |
| | 10 | —— | 10 | —— | 100 | —— | L | |
| | 9 | —— | 11 | —— | 99 | —— | M | |
| | 8 | —— | 12 | —— | 96 | —— | N | |
| | 7 | —— | 13 | —— | 91 | —— | O | |
| Exhauſtion. | 6 | —— | 14 | —— | 84 | —— | P | Indirect debility. |
| | 5 | —— | 15 | —— | 75 | —— | Q | |
| | 4 | —— | 16 | —— | 64 | —— | R | |
| | 3 | —— | 17 | —— | 51 | —— | S | |
| | 2 | —— | 18 | —— | 36 | —— | T | |
| | 1 | —— | 19 | —— | 19 | —— | U | |
| | 0 | —— | 20 | —— | 0 | death | V | |

From

From (A) to (G) includes thoſe diſeaſes which ariſe from the abſtraction of neceſſary ſtimuli, as ſcurvy, petechiæ ſine febre, &c. and points out the degree of ſtimulus neceſſary to reſtore health.

From (H) to (O) includes thoſe variations which may be conſidered as compatible with health, while the correſponding ſtimuli are applied; but, if inordinate or diſproportionate ſtimuli be applied, in any ſtate of the excitement, diſeaſe may be induced.

From (P) to (V) compriſes the degrees of exauſted excitability, or indirect debility, to the account of which almoſt the whole catalogue muſt be placed; for the diſeaſes ariſing from accumulation often ſuddenly paſs into thoſe of exhauſtion, in conſequence of exceſſive ſtimuli.

From the above ſtatement of this ſyſtem, as far as reſpects the cure of diſeaſes, it will be obvious, that the doſes, as well as the medicines themſelves, muſt be regulated by the ſtate of the excitabity; and that in aſcertaining this ſtate and proportioning the ſtimuli to it, is the only field in which the practitioner can exerciſe his ſkill and judgment.

For an application of theſe opinions to the prevention or cure of particular diſeaſes, we preſume ſuch of our readers who deem this ſyſtem worthy of their farther attention, will naturally have recourſe to the author of it, in Dr. BEDDOES's edition of the "*Elements of Medicine*," 2 vols. 8vo. London, 1795; or to "*A View of the Science of Life*," by YATES and MACLEAN, 8vo. Calcutta, 1797. In theſe works, the errors and defects of Brown's ſyſtem are pointed out, and corrected or ſupplied.

